# A quantitative analysis of chronic exposure of selected heavy metals in a model diet in a CKD hotspot in Sri Lanka

**DOI:** 10.1186/s12882-019-1371-5

**Published:** 2019-06-07

**Authors:** T. B. Ananda Jayalal, T. W. M. A. Jayaruwan Bandara, Sanath T. C. Mahawithanage, M. A. Jagath Wansapala, Sapthala P. L. Galappaththi

**Affiliations:** 1 Environmental Health Occupational Health and Food Safety unit, Ministry of Health, Nutrition and Indigenous Medicine, Colombo, Sri Lanka; 2 Ministry of Health, Nutrition and Indigenous Medicine, Colombo, Sri Lanka; 3 Biochemistry; Faculty of Medical Sciences, University of Sri Jayewardenepura, Nugegoda, Sri Lanka; 4 Food Science and Technology, University of Sri Jayewardenepura, Nugegoda, Sri Lanka

**Keywords:** Chronic kidney disease, CKDu, Hypertension, Low IQ, Neoplasms, Low birth weight, Cadmium, Lead, Arsenic

## Abstract

**Background:**

Evidence of chronic low levels of exposure to heavy metals in Sri Lanka has emerged in a number of studies carried out in the recent past. The source and magnitude of such exposures have to be understood in order to assess the risk of adverse health effects of this exposure and to propose suitable public health interventions.

**Methods:**

An assessment was carried out to quantify chronic exposure to cadmium, lead and arsenic through food in people living in an area in Sri Lanka, where prevalence of Chronic Kidney Disease of unknown origin (CKDu) is highest. First a dietary survey was carried out in the subjects to estimate the type and quantity of typical food items in order to estimate an average consumption. A model diet was formulated using this dietary consumption data; and this was thereafter used for estimation of chronic dietary exposure of selected contaminants. In parallel, the levels of contaminants of interest in the various identified food items: cadmium, lead and arsenic, were determined.

**Results:**

Assuming the major route of intake is food, and based on the quantity and type of food items consumed, a 60 kg man is exposed to average doses of 83.7 μg cadmium, 924.1 μg lead, and 180.3 μg arsenic per week. The impact of chronic lead exposure was affirmed by a mean blood lead level of 3.0 μg/dL, with a maximum level of 8.8 μg/dL being observed in some cases.

**Conclusions:**

Chronic low dose exposure of lead from food appears to be a public health concern in the studied population. Cadmium exposure through food appears to be of concern also. However, arsenic exposure through food appears to be within safe limits. As there are numbers of possible adverse health outcomes that can be associated with such estimated exposures of heavy metals, public health interventions are warranted to limit the described harmful exposures. Advocacy on dietary patterns and agronomic practices to lower the contaminants identified are the two broad strategies suggested.

## Background

Evidence of chronic low levels of exposure to heavy metals from food in Sri Lanka has emerged in a number of studies carried out in the recent past [1–7]. With the emergence of chronic kidney disease of unknown origin in certain parts of Sri Lanka since the 1990s, a number of studies have been conducted involving the analysis of environmental, food and biological samples, and these have pointed to chronic low-level exposure of heavy metal contamination as being among the main postulated risk factors.

A considerable volume of credible data is now available with regard to these contaminants in various constituents of environmental, food and biological samples [1, 2, 6–8]. However, an objective, science-based public health intervention to reduce these risk factors has not yet been formulated as clarity has yet to emerge on the magnitude and chronic exposure of the postulated risk factors.

Human exposure to toxic heavy metal is a serious public health concern in most developed countries. However, Sri Lanka being a tropical small island nation with low economic status and limited research capabilities, which has not yet satisfactorily estimated the total exposure to these toxic metals and therefore not provided adequate emphasis on, or highlighted the associated risks to public health. Moreover, dietary habits of Sri Lankans in general are distinctly different from most of its global counterparts [9], thereby warranting a deeper evaluation of the situation. Sri Lanka is categorized in the G14 cluster of global dietary consumption groups according the WHO GEMS diet categorizations [9]. Other countries included in this cluster are: Comoros, Fiji Islands, Kiribati, Papua New Guinea, Solomon Islands and Vanuatu. A common feature among this cluster of countries is the high consumption of cereals, especially rice, in their diets. Because of this peculiar food preference and habits, a reasonable evaluation of dietary pattern and associated exposure to heavy metals through food needs priority attention.

Chronic exposure to cadmium had been incriminated in chronic renal failure among other health outcomes [10]. Cadmium exposure through food had been identified as the main mode of exposure in non-occupational settings [11]. Common mechanisms in nephropathy induced by toxic metals are described by Sabolić I. [12]. Dietary cadmium intake had been identified as one of the main routes of entry into the human body.

An increased risk of nephropathy was noted in workers with lead blood levels over 3.0 μmol/L [13]. The European Union Food Safety Agency has developed models for chronic lead exposure from food and resultant blood lead levels, and quantified the corresponding increase in systolic blood pressure, the corresponding decrease in glomerular filtration rate and intelligent quotient recently [14].

Chronic low-level exposure to lead and cadmium have been hypothesized as risk factors in not only CKDu but also in neurodevelopmental defects in children, hypertension in adults, neoplasms, low birth weight and malnutrition in various studies [15–22]. Further synergistic effect of cadmium and lead on nephrotoxicity has been described in the scientific literature [9, 23].

Arsenic has also received attention as a potential causative agent of CKD in scientific literature and the popular press [24, 25].

A case control study [1] comparing CKDu affected population and healthy controls, have shown statistically significant high urine excretion of cadmium in CKDu patients.

Lead and cadmium have also been detected in food samples [1–8] but comprehensive exposure assessment has not been done in those studies perhaps it was not part of the study objectives. Blood lead levels in similarly affected people which indicate recent exposure had been reported [6]. However, bone lead, which indicates long term exposure had not been done in the available studies to preclude or establish the long term exposure.

An area with the highest prevalence of CKD was selected for the study so as to capture the potentially worst-case scenario. From the possible sources of exposure, we focused on food since the existing evidence suggests that other occupational exposures, e.g. through air, is far less likely in the selected area/s. Further, there is no evidence that potable water is a significant source of contamination by the selected heavy metals [26]. To calculate the possible exposure through food, it was decided to develop a model diet by means of a dietary survey from which estimations of total exposure against time can be estimated once the magnitude of contamination of food by the selected heavy metals is known.

Among the heavy metals of concern, cadmium and lead were selected for the study as they are known to cause chronic kidney damage. Arsenic was also considered for the study as there is speculation that arsenic may also be implicated in the causation of CKDu in Sri Lanka.

This study is a quantitative analysis of chronic exposure to selected heavy metals, (lead, cadmium and arsenic) via typical food items consumed by residents in areas where the prevalence of Chronic Kidney Disease of uncertain aetiology (CKDu) is highest.

The objectives of this study were to:

1. Determine food consumption patterns of diagnosed Chronic Kidney Disease (CKD) patients living in an area where the prevalence of CKDu is highest. (Medawachchiya Medical Officer of Health area).
2. Determine lead, cadmium and arsenic levels in commonly consumed raw food items from randomly selected households with confirmed CKD patients living in two MOH areas where the prevalence of CKDu is highest in Sri Lanka, (Medawachchiya and Padaviya).
3. Quantify the levels of exposure to cadmium, arsenic and lead through food in individuals living in randomly selected households with CKDu patients.
4. Determine blood lead levels and prevalence of lead poisoning symptoms of the individuals living in selected households with CKD patients.

## Methods

### Determination of the food consumption pattern in diagnosed chronic kidney disease (CKD) patients living in a MOH area with the highest prevalence of CKDu. (Medawachchiya)

#### Selection criteria

**Fig. 1:**
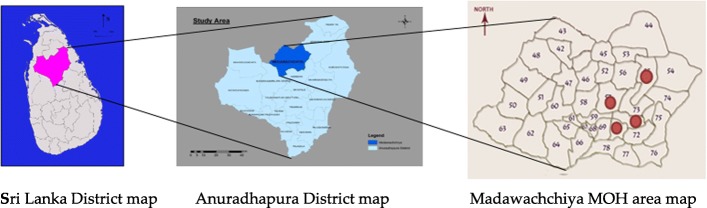
Study Area; Map of Sri Lanka Anuradhapura District, and Map of Medawachchiya MOH area

A total number of 77 CKD patients were recruited from the Medawachchiya MOH (Medical Officer of Health) area which shows the highest prevalence of CKDu in Sri Lanka [27]. The MOH area comprises 37 Grama Niladari (Village Headman) (GN) areas which were ranked by the prevalence of CKDu, with four being randomly selected from the top 10 divisions (Fig. 1 - depicted in red). Each GN area comprises 2–3 villages and the village with highest prevalence from each GN area was selected. Almost all the patients diagnosed with CKD from each selected village were recruited for the survey using the lists available with the Grama Niladhari. Diagnosis of CKD was confirmed by inspecting the medical records of the study subjects.

#### Dietary survey procedure

The dietary survey was carried out using a semi-quantitative food frequency questionnaire (FFQ) during the period August to September 2016. The FFQ was formulated using commonly consumed food items identified in a previous survey [28]. Subjects were interviewed on qualitative and quantitative food consumption and the data duly recorded. The review included type, approximate quantity (per familiar household measuring devices such as coconut shells, tablespoons etc.) and frequency of food intake over specific time periods. The questionnaire covered the past 1-month’s consumption pattern.

Portion sizes of each food item were measured in the laboratory and estimations were thereafter made to build the model diet.

To determine the intake of a food item by a patient, the average portion size was multiplied by its frequency of consumption by the patient.

$$ Patient^{\prime }s\  intake\ of\ a\  food\ item\kern0.5em = Portion\ size\ast Frequency\ of\ consumption\ of\kern0.5em the\ food\ item $$

Finally, the mean intake of commonly consumed food types per week by the population was calculated.

Sri Lankan dietary habits do not show seasonal changes in general and it was assumed that dietary pattern does not show considerable seasonal variation in the study population.

### Determination of lead, cadmium and arsenic levels in commonly consumed raw food items from randomly selected households with CKDu patients living in Madawachchiya and Padaviya MOH areas

#### Selection criteria

A total of 104 households with patients diagnosed with CKD were randomly selected from the Madawachchiya and Padaviya MOH areas which are considered as areas with the highest prevalence of CKD patients [29]. Madawachchiya MOH area is shown in the Anuradhapura district map in Fig. 1. The Padaviya MOH area is situated northeast of Medawachchiya. The area with the highest prevalence was selected since this type of “hotspot sampling” may help establish stronger links with CKDu risk factors [29]. Selected households were visited and 277 samples were collected from 70 households from the food items available at the time of these visits. In addition, two inland fish samples from the Padaviya tank were collected for analysis.

#### Analysis of samples for heavy metals

Food samples were analysed by ICP MS AOAC official method for arsenic (986.15:2012), cadmium (999.10:2012) and lead (999.11:2012) [30].

About 0.5 g of the homogenized samples were digested using Mars Xpress microwave digester (CEM; Matthews, NC). Digestion was performed with 5 ml - 10 ml HNO_3_ (69.0% extra pure; SRL, India) diluted to 50 ml using deionized water.

Concentration of trace elements arsenic, cadmium and lead were determined using Thermo ICapQ (Thermo-Fisher Scientific Inc., Bremen, Germany) Inductively Coupled Plasma Mass Spectrometry (ICP-MS). Multi-element ICP-MS standards (AccuStandard, USA) were used for the instrumental calibration. Six Standards of 1, 2, 5, 10, 20 and 50 μg/L were used for the calibration. Limit of quantification (LOQ) for HNO_3_ digested analyte were 0.0006 mg/l, 0.0003 mg/l, 0.0004 mg/l for lead, cadmium and arsenic respectively. Analytical results were expressed on wet matter basis.

#### Quantifying the levels of exposure to cadmium, arsenic and lead through food of individuals living in randomly selected households with CKD patients

From the results of dietary survey, and available national data, a model diet for a 60 kg healthy male was developed. The contribution of selected contaminants from consuming the model diet was calculated to estimate the chronic exposure. Most such estimates seen in the scientific literature are based on a standard of a 60 kg male, and the same was followed in this study to facilitate comparisons.

### Determine the blood lead levels of the individuals living in randomly selected households with CKD patients

#### Selection criteria

The inhabitants of same 104 households selected for the above study were used to quantify blood lead. There was 8 months’ time lapse between food sample collection and blood sample collection due to some study limitations. Blood samples were collected from occupants from 92 households (96%), with the remainder not being possible due to vacant occupancy at the time of visit. A review of the symptoms of lead poisoning as described in the Agency for Toxic Substance and Disease Registry [15] was undertaken with each subject using an interviewer administered questionnaire.

#### Sample collection

Blood samples were collected from two occupants from each household. CKDu patients were given priority, while the others were apparently healthy subjects. After obtaining written informed consent from each subject (and their parent or legal guardian in the case of children under 18 years of age), venous blood was drawn and collected in a capillary tube of 50 μl which was then transferred to a treatment reagent tube. An electrochemistry sensor system of Lead Care II blood lead testing system was used to analyse the blood lead (LOQ 3.3 μg/dL). Two known concentrations of a known lead salt in a buffered solution with bovine serum albumin was used as quality control for each batch of test samples (ESA Biosciences Inc. USA).

#### Sample handling and analysis

All the samples were collected in uncontaminated containers and transported to the laboratory on the same day.

All statistical analyses were performed by SPSS ver. 23.0. Concentrations less than the limit of quantification (LOQ) were allocated a value of half-LOQ for subsequent statistical interpretations (USEPA 2000) [31].

Food samples were analyzed for concentration of cadmium, lead and arsenic. The mean, minimum and maximum values were calculated. Similarly, blood lead mean, minimum and maximum values were calculated.

### Ethical considerations

The study was conducted in accordance with the Declaration of Helsinki. The protocol was approved by the Ethics Committee of the Medical Research Institute, Sri Lanka (Project No. 42/2016) for analysis of heavy metals in food items and Sri Lanka Medical Association (ERC/17–011) for the blood lead analysis. Informed prior consent was obtained in written format from each subject and additional written proxy consent obtained from the subjects if they were under 18 years of age for inclusion in to the study.

## Results

### Food consumption pattern of diagnosed chronic kidney disease (CKD) patients living in a medical officer of health area where the CKDu is high (Medawachchiya)

Food consumption patterns were studied in 77 patients with CKD. All patients were previously diagnosed with CKD with eGFR (estimated Glomerular Filtration Rate) being equal to or less than 60 ml/min by CKD EPI formula. Those who were terminally ill were not included in the study. All patients were over 40 years of age and 57% of them were male. Rice, vegetable and inland fish were the top three food categories consumed, and their average consumptions are given in Table 1. The table also gives the national and district consumption data based on the latest national survey carried out [32]. Consumption of all other food varieties were negligible, which in itself is a very significant observation. The low levels of inland fish and other sources of animal protein consumed were consistent with their regular practice and these items were not deliberately reduced based on medical advice to lower protein intake due to their disease.

**Table 1:** Consumption of major food items of CKD patients in relation to district and National intake data

Food items /Groups	Average consumption per person per week (kg) in CKDu hotspot		Average per capita intake per week (kg)^a^	
			Anuradhapura District	National
	Mean	SD	Mean	Mean
Rice	2.50	1.2	2.3	2.2
Vegetables	0.79	0.3	Not available	1.0
Inland Fish	0.06	0.07	Not available	Not available

^a^(Household Income and Expenditure Survey 2012/13, Department of Census and Statistics, Ministry of Policy Planning Economic Affairs, Child Youth and Cultural Affairs Sri Lanka) [32]

### Lead, cadmium and arsenic levels in raw food items commonly consumed in randomly selected households with CKD patients in two medical officers of health areas where the prevalence of CKDu is highest (Medawachchiya and Padaviya MOH areas)

Food items available at the households at the time of visit were collected and analysed for lead, cadmium and arsenic levels. A total number of 277 food samples were analysed. For the purpose of comparison with international values, criteria prescribed by European Union Categorization (nine categories) [33] were adopted. Rice was considered as a distinctly separate entity from other cereals as it was the main staple food collected as samples except for the one sample of finger millet (Kurakkan).

Whether the source of each food item was from their own cultivation plots (self-grown) or from elsewhere was also noted (Table 2). Rice was available at almost all households. Long beans (*Vigna unguiculata ssp. sesquipedalis*) and manioc tubers (*Manihot esculenta*) were the most predominant samples at households after rice. Gotukola (*Centella asiatica*) was the main item among 44 samples of leafy vegetables found at households. Long bean, winged bean (*Psophocarpus tetragonolobus*) and cowpea (*Vigna unguiculata*) were the main legumes found and were present only in 19 households. There were eight types of fruit, lime (*Citrus aurantiifolia*) being the predominant item in 10 households. Only negligible amounts of other food categories were found in the households surveyed.

**Table 2:** Food Groups and Respective Food Items

Food Group	Food item/s	Total Number of samples	Number of self-grown samples (%)
Rice	Rice (*Oryza sativa)*	65	34 (52)
Fruit/ Flower Vegetables	Chilli (*Capsicum annuum spp*)	9	7 (78)
	Banana Blossom (*Musa acuminate spp.*)	9	4 (44)
	Moringa (Moringa *oleifera*)	8	5 (63)
	Thibbatu (*Solanum torvum)*	8	5 (63)
	Elabatu (*Solanum indica*)	7	5 (71)
	Brinjal (*Solanum melongena*)	6	4 (67)
	Amberalla (*Spondias dulcis*)	6	4 (67)
	Capsicum (*Capsicum annuum spp*)*.*	5	4 (80)
	Kakiri (*Cucumis meloi*)	5	4 (80)
	Okra (*Abelmoschus esculentus*)	4	3 (75)
	Pumpkin (*Cucurbita maxima*)	2	1 (50)
	Ridge Gourd (*Luffa aegyptiaca*)	2	0 (0)
	Kochchi (*Capsicum frutescens*)	6	5 (83)
	Ash Plantain (*Musa paradisiaca*)	1	1 (100)
	Biling Fruit (*Averrhoa bilimbi*)	2	1 (50)
	Bitter Gourd (*Momordica charantia*)	1	0 (0)
	Breadfruit (*Artocarpus altilis*)	1	1 (100)
	Jackfruit (*Artocarpus heterophyllus*)	1	1 (100)
	Seegiri Batu (*Solanum virginianum*)	1	1 (100)
	Tomato (*Solanum lycopersicum*)	1	1 (100)
Leafy Vegetables	Gotukola (*Centella asiatica*)	14	7 (50)
	Spinach (*Spinacia oleracea*)	7	3 (43)
	Thampala (*Amaranthus gangeticus*)	7	6 (86)
	Kathurumurunga (*Sesbania grandiflora*)	5	3 (60)
	Kankun (*Ipomoea aquatica*)	3	2 (67)
	Anguna Kola (*Wattakaka volubilis*)	2	1 (50)
	Mukunuwenna (*Alternanthera sessilis*)	2	1(50)
	Cassava/Manioc Leaves (*Manihot esculenta*)	2	1 (50)
	Moringa Leaves (*Moringa oleifera*)	1	0 (0)
	Curry Leaves (*Murraya koenigii*)	1	1(100)
Legume Vegetables	Long Beans (*Vigna unguiculata ssp. Sesquipedalis*)	15	13 (87)
	Winged Bean (*Psophocarpus tetragonolobus*)	2	2 (100)
	Cowpea (*Vigna unguiculata*)	2	2 (100)
Fruits	Lime (*Citrus aurantiifolia*)	10	7 (70)
	Banana (*Musa spp.*)	4	2 (50)
	Mango (*Mangifera indica*)	2	0 (0)
	Sour Sop (*Annona muricatai*)	3	1(33)
	Passion Fruit (*Passiflora edulis*)	2	1(50)
	Orange (*Citrus sinensis*)	2	1(50)
	Belli Fruit (*Aegle marmelos*)	1	0 (0)
	Pomegranate (*Punica granatum*	1	0 (0)
Fats and Oil	Coconut (*Cocos nucifera*)	5	1 (20)
	Sesame (*Sesamum indicum*)	1	1 (100)
Cereal (non- rice)	Kurakkan (*Eleusine coracana*)	1	1 (100)
Egg	Egg	2	1 (50)
Inland Fish	Inland Fish	2	0 (0)
Root & Tuber Vegetables	Manioc (*Manihot esculenta*)	15	9 (60)
	Kiri Ala (*Xanthosoma sagittifolium*)	9	4 (44)
	Hulan Keeriya (*Maranta arundinacea*)	1	1 (100)
	Beet (*Beta vulgaris spp.*)	1	0 (0)
	Kohila (*Lasia spinosa*)	1	1 (100)
	Ratala (*Solenostemon rotundifolius*)	1	1 (100)
Total		277	165 (60)

Sixty percent (60%) of food items (*n* = 165) collected were from their own cultivation (self-grown), with the rest being purchased from nearby weekly fairs or other outlets. This indicates that those afflicted with CKDu consume food mostly from their own farms or produce from nearby or proximal farms, and this may in turn indirectly support the hypothesis for the geographical localization of some of the adverse health effects described in this article.

Approximately 80% of the samples tested (219 out of 277) were found to have detectable levels of lead. The mean lead content of 277 samples was 216 μg/kg ± SD 223. Only 25 food items (9%) contained a cadmium level above the LOQ for Cadmium from 31 to 93 μg/kg, with a mean of 18 μg/kg. Out of 65 samples (15%) of rice, 10 showed levels of cadmium above the LOQ. The mean cadmium level in rice was 22 μg/kg ± SD 19. Thirty (11%) samples out of 277 showed levels above LOQ for arsenic ranging from 40 to 1108 μg/kg with a mean of 41.09 μg/kg ± SD 111.48 μg/kg and only 10 (1%) samples had the levels above 100 μg/kg. (Table 3). Three (5%) rice samples (out of 65) contained > 100 μg/kg of Arsenic. Table 4 depicts the lead, cadmium and arsenic concentrations of categories of food items available at households.

**Table 3:** Descriptive data of all the food samples

	Minimum μg/kg	Maximum μg/kg	Percentage of sample above LOQ
Lead	61	1780	80
Cadmium	31	93	9
Arsenic	41	1108	11

**Table 4:** Lead, cadmium and Arsenic concentrations of food items available at households

Food Group		Lead Concentrations (μg/kg)		Cadmium concentrations (μg/kg)		Arsenic Concentrations (μg/kg)	
	n	Mean	SD	Mean	SD	Mean	SD
Rice	65	236.3	235.7	21.9	18.7	45.5	107.7
Fruit/Flower Vegetables	88	232.1	282.4	17.8	12.4	44.9	115.1
Leafy Vegetables	43	212.4	174.6	18.8	8.9	66.4	187.1
Legume Vegetables	19	184.5	145.7	18.9	6.8	20.0	0.0
Fruits	24	187.9	157.2	15.0	0	20.0	0.0
Fats and Oil	6	154.3	154.9	27.0	21.5	20.0	0.0
Inland Fish^a^	2	57.0	38.2	15.0	0.0	20.0	0.0
Root & Tuber Vegetables	27	185.5	164.8	15.0	0.0	20.0	0.0

^a^Inland fish were included here as it is an important constituent in the diet, however not subjected to statistical test due to inadequate sample size

The Kruskal-Wallis test was performed to test whether there is a statistical difference between mean values of lead, cadmium and arsenic contents in food categories which had at least 6 samples. (This includes 272 samples excluding egg and cereals). In the instances where the Kruskal-Wallis test showed that statistical differences did exist among categories, the Mann-Whitney U test was applied to test the statistical significance among each two groups of food categories.

**Fig. 2:**
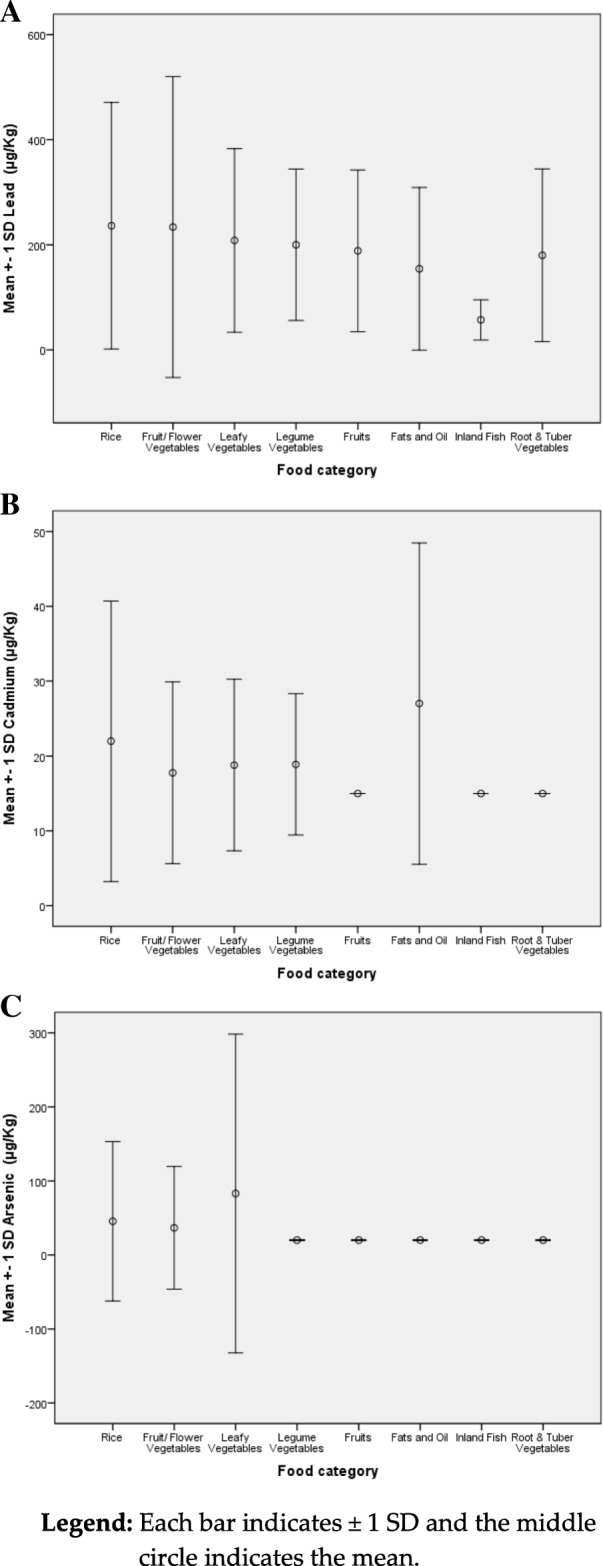
**a**: Lead levels of selected food categories. **b**: Cadmium levels of selected food categories. **c**: Arsenic levels of selected food categories

Results for important food categories are shown in Fig. 2 a, 2 b, and 2 c.

When the Kruskal-Wallis test is done on the values of Fig. 2a, there was no significant difference between food category groups for mean lead content. (*p* = 0.668), indicating that all the food categories are equally contaminated with lead except inland fish. Even though the mean value for inland fish shows a conspicuous difference, the sample size limitation for this category does not allow statistical testing.

When the Kruskal-Wallis test is applied to values of Fig. 2b, it showed a significant difference between groups for mean cadmium. When the Mann-Whitney U test is applied, the differences of the mean values between rice and fruits (*p* = 0.039), rice and root vegetables (*p* = 0.029) were found to be statistically significant. The difference between mean values of cadmium in Fat & oils and flower vegetables were also statistically significant (*p* = 0.018). Mean values of cadmium in Fruits versus legume vegetables (*p* = 0.042) and root vegetables (*p* = 0.032) also indicated significant differences. Another significant difference was reported between fruits and fats & oil categories (*p* = 0.003). Also, Fats & Oil category and Root & Tuber Vegetables (*p* = 0.002) demonstrate a significant difference in their mean cadmium concentrations. Even though the mean value for cadmium in inland fish shows conspicuous differences, again the sample size limitation for this category does not allow such statistical testing.

The mean level for cadmium in rice in our study was 22 μg/kg [SD 19 μg/kg]. However, a mean cadmium concentration of 117 μg /kg in rice from Medirigiriya which is also a CKDu hotspot, has been reported by Levine et al. (2016) [6]. Similarly, considerably higher mean values of cadmium in rice, than found in our study have been reported in a number of other studies in Sri Lanka [1–7, 34].

When the Kruskal-Wallis test is applied to values of Fig. 2c, a statistically significant difference of mean arsenic concentrations among food categories were found. When Mann-Whitney U test is done for each two groups, there were significant differences between the following. Statistically significant differences in arsenic concentrations were found between rice and vegetables (*p* = 0.008); rice and legume vegetables (*p* = 0.018); rice and fruits (p = 0. 008); rice and root & tube vegetables (*p* = 0.005); leafy vegetables and fruits (*p* = 0.039); and between leafy vegetables and root & tuber vegetables (*p* = 0.029). No statistically significant differences in arsenic concentrations were found between other food categories. Again, even though the mean value of arsenic in inland fish shows conspicuous differences, the sample size does not allow such statistical testing.

### Mean values of lead, cadmium and arsenic concentrations in the food

Mean values of the above contaminants in rice and vegetables (which comprise the main diet of the study population) and which can cause considerable health impacts, are shown in Table 5.

**Table 5:** Mean values of lead, cadmium and arsenic in rice and commonly consumed vegetables

Heavy Metal	Mean Value in Rice (μg/kg)	Mean Value in Vegetables (μg/kg)
lead	236.3	215.2
cadmium	22.0	17.7
arsenic	45.5	43.8

### Blood lead levels of the individuals living in the randomly selected households with CKD patients

A Total of randomly selected 92 Households having at least one CKDu patient from the Padaviya and Medawachchiya MOH areas were visited, 184 residents (approximately 2 people representing each household) were recruited and their blood lead levels were tested. Blood lead levels by different socio-demographic characteristics are presented in Table 6.

**Table 6:** Blood lead; Descriptive statistics on subject groups

Variable		Blood Lead Status (μg/dL)			
		n	Mean	SD	P- Value (Mann- Whitney Test)
Status of CKDu	Positive	103	3.1	1.8	0.184
	Negative	81	2.8	1.7	
Area of Residence	Padaviya	80	3.1	1.7	0.279
	Medawachchiya	104	2.9	1.7	
Sex	Male	76	3.2	1.9	0.143
	Female	108	2.8	1.6	
Age	≤40	85	2.8	1.7	0.046
	> 40	99	3.2	1.7	
Total		184	3.0	1.7	–

Forty two percent (42%) of study population showed detectable levels of lead levels in their blood (LOQ 3.3 μg/dL). The value ranged from 1.7 (extrapolated) to 8.8 μg/dL with a mean of 3.0 μg/dL + SD 1.7.

Table 6 gives information on subjects by CKDu status and socio-demographic factors as well as information on descriptive statistics of each group. Blood lead levels of persons above 40 years of age shows significantly higher values than the persons below 40 years old when the Mann-Whitney U test is applied, indicating prolonged exposure to lead. Among the socio-demographic factors studied, only age had a statistically significant (*p* = 0.046) difference.

### Status of chronic lead poisoning symptoms

Chronic lead poisoning symptoms described in the Agency for Toxic Substance and Disease Registry of USA [15] reported among subjects the following: Thirty five percent of respondents reported no symptoms. Fifty one percent reported 1–5 symptoms and 15% reported 6–11 symptoms. Out of 65% who reported symptoms, the following were found (Table 7).

**Table 7:** Prevalence of symptoms associated with lead poisoning

Symptom	Percentage reported
Joint and muscle pain	40
Fatigue and Irritability	35
Dyspepsia	34
Numbness or tingling in the extremities	26
Headache	25
Constipation	15
Difficulties with memory or concentration	14
Hearing loss	14
Loss of appetite	14
Sleep problems	14
Abdominal pain	7
Nausea	5
Colic	3
Seizures	2
Mood disorders	1
Wrist/foot drop	0

However, all the above symptoms cannot be attributed directly to chronic lead poisoning since some of the symptoms are common for CKD patients. Therefore, this data does not indicate the extent or magnitude of lead exposure. However, this data may be useful in subsequent follow up studies.

## Discussion

The effects of chronic exposure to a food contaminant depends mainly on the amount consumed (the dose response effect). Thus, food consumption data and the degree of contamination of the food by the contaminant in question are required for a reasonable estimation of chronic exposure. This study aims to fill this gap in information in this important area of public health.

The authors of this study adopted the approach of a “total diet study” method which is the recommended basis of exposure estimation to the contaminants in question. Further, it was carried out in a CKDu hotspot. A dietary survey coupled with food analysis was carried out. Blood lead was studied in the same exposed population to further confirm the finding of exposure estimation, as it was the heavy metal of highest concern in the study.

### Diet survey

It was found that rice, vegetables and inland fish comprise the major dietary components in the study population. The average consumption of rice as estimated is higher than the published district and national per capita average consumption levels as well as the WHO G14 cluster of global dietary consumption group for cereals. Rice was also found to comprise the largest component by weight in the diet in the study cohort. It should be noted that the district and national averages are derived from studies which include the entire population, which include children and the elderly, who consume smaller quantities, while the subjects in this study were mostly CKD patients who were also over 40 years of age. Further, the subjects admitted to consuming less food since they fell ill (their sickness tended to depress appetite) and admitted to consuming greater quantities when they were well. The authors believe that the uniquely high consumption levels of rice in the study population offer a credible basis to embark on further related studies aimed at deriving potential causation of CKDu from chronic exposure to heavy metals. Vegetable consumption levels appear to be marginally lower than both the district and national averages.

Commonly consumed vegetables were fruit vegetables, pulses, roots/tubers, starchy vegetables, green leafy vegetables and, Leguminous pods.

The survey revealed very low consumption levels (60 g) of inland fish per week in this population. Allinson G et.al and Premarathna HL et.al, also have previously described that freshwater fish consumption is not a crucial dietary component in the study population [34–36].

Males were found to consume significantly higher amounts of inland fish than their female counterparts (*p* = 0.017). There was no significant statistical difference between consumption of rice and vegetables between males and females.

The main source of income in the study cohort is agriculture; and the resultant low income, poor infrastructure facilities and rural living conditions may serve to explain the amounts and limited range of food types by this population.

The study population was found to consume food mostly from their own farms (65%) or food purchased from nearby village markets which sell produce from nearby farms, and which may in effect contain similar levels of toxic heavy metals. This finding may indirectly support the observed geographical localization of the Chronic Kidney Disease (CKDu hot spots). Further it can be argued, if the source of the contamination of the food is not aggressively controlled, there is danger that the adverse health effects of those contaminants may spread from its boundaries and in due course cause similar adverse effects on the wider population of Sri Lanka, including the urban regions.

### Food analysis for selected heavy metals

The data shows that rice is contaminated with both lead and cadmium in considerable number of samples and lead is a contaminant found in almost all food items in the study area. Inland fish (albeit in the small sample size studied) appears to be comparatively safer than all other foods in this regard.

### Variability of data of determined concentration of selected heavy metals

All three metals analysed show wide variance, leading to SD values being very close to the mean value. It indicates substantial proportion of samples are heavily contaminated than others.

### Determination of model diet and exposure assessment

For the exposure assessment, methodology used by Joint WHO/FAO committee on food additives (JEFCA) and European Food Safety Authority (EFSA) panel on Contaminants in the Food Chain (CONTAM) was adopted.

For the purpose of calculation of chronic total exposure, our diet survey and national data available from other sources were taken in to consideration and a model diet for 60 kg man was presumed: A diet of 3 kg of rice with 1 kg of vegetables per week was assumed to be consumed by a healthy man weighing 60 kg living in the study area as the model for exposure calculation. Mean lead, cadmium and arsenic levels in the food commodities consumed by the subjects are used for the calculation of exposure of the people living in the area.

If the rice and vegetable lead values were taken in to consideration a 60 kg man is exposed to 236.3 μg × 3 kg + 215.2 μg x1kg = 924.1 μg of lead per week. Contribution from other food varieties, tobacco smoking, water, air, dust and soil might conceivably add a few additional micrograms of lead to this value.

Effect of above exposure was further established by the blood lead study of exposed subjects; Forty two percent (42%) of study population had detectable levels of lead in their blood (LOQ 3.3 μg/dL). The value ranged from 1.7 (extrapolated) to 8.8 μg/dL with a mean of 3.0 μg/dL + SD1.7. Similar blood lead levels ranging from 1.03 to 9.09 μg/dL (minimum and maximum respectively) with a mean of 3.6 μg/dL were found in a comparative study population in Medirigiriya in Sri Lanka [6].

Until 2011, JECFA considered the Provisional Tolerable Weekly Intake (PTWI) for lead as 25 μg/kg body weight (bw), which is equivalent to 1500 μg for a 60 kg person. However, in 2011, JECFA reported that a PTWI of 25 μg/kg bw was associated with a decrease of at least 3 IQ points in children and an increase in systolic blood pressure of approximately 3 mmHg in adults; and concluded that the PTWI of 25 μg/kg bw can no longer be considered as being appropriate to protect health. This resulted in the previously recommended PTWI [11] being withdrawn.

The mean chronic exposure to lead in the study populations is estimated as 62% of the pre-2011 JECFA PTWI, pointing to insufficient protection from lead. As this is a mean value it can be further concluded that in a certain segment of the study population, chronic exposure to lead is a matter for greater concern considering the recent scientific conclusions of health effects of such exposures. The detected lead levels are known to cause decreased IQ levels in children as well as increasing systolic blood pressure and reduction of GFR (Glomerular Filtration rate) in adults, which increases the public health significance of the finding. If the JECFA limit of lead exposure is applied to the study population, it can be assumed that something close to 3 IQ points decrease in children and close to 3 mmHg systolic blood pressure increase can be predicted in the study population.

The detection of blood lead in the subjects consuming contaminated food types strongly suggest the presence of bioavailable lead in food. Lead is a known heavy metal toxin that crosses the placenta and blood-brain barrier, depositing in foetal tissues. The evidence on neurological consequences of prenatal exposure to lead appears to reflect changes in cognitive impairment. Primary prevention of lead exposure across the entire human life span is being recommended in current scientific literature [16].

The phenomenon of fluoride being known to increase the absorption of lead may be playing a synergistic role in this situation as it had been reported that fluoride concentration in water in the CKDu affected areas is higher [37].

The European Food safety Agency (EFSA) Panel on Contaminants in the Food Chain (CONTAM) considers developmental neurotoxicity, systolic blood pressure increases and chronic kidney disease as the most potent adverse effects of lead [14]. Dose response modelling for these adverse effects was used to determine the 95th percentile lower confidence limit of the benchmark dose (BMD) of 1% extra risk. The respective BMDLs derived from blood lead levels and corresponding dietary intake values for developmental neurotoxicity, BMDL_01_, are 1.2 μg/dL and 3.50 μg/kg body weight per week respectively. If these values are applied to the population of our study, they would be predicted to result in a lowering of 2.5 and 4.3% of full-scale Intelligence Quotient (IQ) when the average blood lead levels and corresponding dietary intake is considered respectively. Here it is assumed that exposure levels of children are similar to adults, when in fact exposure of children may actually be higher due to their lower body weight, thereby placing them at a higher risk than estimated.

The CONTAM panel has determined the 95th percentile lower confidence limit of the benchmark dose (BMD) of 1% mean annual increase of population based systolic blood pressure BMDL_01_, as a blood lead level of 3.6 μg/dL, which corresponds to dietary intake values in μg/kg body weight per week of 10.50 μg. If these values are applied to the population of this study, levels of 1.2 and 1.5% annual increase of population based systolic blood pressure can be derived. Generally, such an increase in populations is associated with a significant risk of stroke and other cardiovascular events.

The CONTAM panel has determined the 95th percentile lower confidence limit of the benchmark dose (BMD) of 10% change in the prevalence of CKD defined by GFR below 60 mL/ 1.73m^2^ body surface per minute, BMDL_10_, as 1.5 μg/dL for blood lead and a corresponding dietary intake of lead equal to 4.41 μg/kg body weight per week. If these values are applied to the population of our study, they are at risk of 20% and 36% reduction in GFR respectively when the average blood lead levels and dietary intake of lead exposure is considered.

Retarded neurodevelopment in children of Sri Lanka as a consequence of exposure to lead emerges as a serious concern as the average Intelligence Quotient (IQ) of the Sri Lankan population is 79, which positions it below 131 countries according to the IQ Research [38].

In 2016 low birth weight (LBW) was recorded at 16%, underweight among children less than 5 years old at 20.5%, wasting at 15.1% and stunting at 17.3% in Sri Lanka [32]. Ischemic heart disease and neoplasms are among the 10 leading causes of hospitalization and death [39]. Thirty percent of the 35 to 64-year-old Sri Lankans are hypertensive [40]. All these indicators point to significant health concerns with consequential societal, economic and other adverse effects. In addition to the above health effects, a number of studies have shown that lead and cadmium exposure is associated with low birth weight [17].

The mean total cadmium exposure levels determined in the model diet is 22.0 μg × 3 kg of rice + 17.7 μg × 1 kg of vegetables = 83.7 μg per week. Other food, water and other sources may contribute an additional exposure to cadmium amounting to a few micrograms. The corresponding tolerable weekly intake (PTWI) is 152 μg per week for a 60 kg man [15]. This indicates that the study population is within the accepted tolerable limits when mean exposure from food only considered.

However, previous studies have shown higher concentrations of cadmium: [1–8]. A number of possible explanations can be given for this observed discrepancy, namely: the samples have been collected at different periods of time and from different sources, different analytical methods may have been used, and there may be seasonal variation in contaminants found in the commodities. In our study food samples were collected from affected households where the sampled food was retained for their own consumption. In contrast, some of above studies used samples sourced from the market.

The main health effects of chronic exposure to cadmium is nephrotoxicity, malignancy and association with low birth weight. Cadmium also appears to be associated with overall cancer mortality in men and women [10, 18, 19]. Multivariate models have established relationships between blood cadmium tertials and foetal growth parameters; namely birth weight, low birth weight, birth weight percentile by gestational age, small for gestational age, pre-term birth, length, and head circumference [20–22].

Chronic total exposure of arsenic appears to be of 45.5 μg X 3 + 43.8 μg × 1 = 180.3 μg per week in a 60 kg man. Provisional tolerable weekly intake (PTWI) recommended for arsenic by JECFA until 2011 was 15 μg per kg body weight which is equal to 900 μg for a 60 kg man. The study population is exposed to 20% of the tolerable intake showing arsenic exposure in the study population seems not to pose a significant health risk. However, in 2011 JECFA has withdrawn the PTWI of 15 μg per kg body weight stating that this guideline is no longer appropriate, as such exposure falls within the Benchmark dose (BMDL) of a 0.5% increase in incidence of lung cancer [41]. However, as the estimated exposure of arsenic is five times less in the study population, it can be assumed safely that the increase in incidence of lung cancer may be very much lower than 0.5% from arsenic exposure through food in our study population.

## Conclusions

The diet survey of this study revealed the restricted consumption pattern and food habits of the study population and geographical restriction of their source of food to the area where they live. They consume considerable amounts of rice, 2.5 kg (SD 1.2 kg) and 0.79 kg (SD 0.3 kg) of vegetables per week. Consumption of other varieties of food including food of animal origin are minimal. This poses two kinds of health threats: the diet while being adequate in calories, is deficient in protein and micronutrients; and repetitive consumption of the same kind of food over long periods could lead to chronic exposure to toxic substances found in those food commodities. This seems to be a concern in the general population especially for contaminants like cadmium and lead which have long half-life in the human body, which poses known health risks; as well as for the unborn, where foetuses are exposed to these adverse impacts in utero from the point of conception.

The study has revealed that lead contamination was found in all the food groups tested; rice, vegetables, leafy vegetables, legume vegetables, fruits and root vegetables which comprise the major constituents of the diet in people living in those households. The total average lead exposure is 962 μg per week from food commodities. This would seem to pose a serious public health threat in the people living in the area as it correlates with considerable blood lead levels detected in the exposed population. Chronic low-level lead exposure is known to cause neurodevelopmental effects leading to lowering the IQ in children, hypertension and lead nephropathy in adults.

In this study cadmium was detected in considerable quantities in rice but at lower levels than in the previous studies. Except for the rice other food commodities tested do not contribute to chronic cadmium exposure substantially. The cadmium levels reported in this study are lower than the most of the earlier studies carried out in the region and elsewhere in Sri Lanka. However, the reported mean value plus one SD considered for the exposure calculation model shows that the tolerable weekly intake of 2.52 μg per kg BW per week is exceeded in 16% of population.

The authors note that a synergistic effect between cadmium and lead leading to nephrotoxicity has been described in the literature [11]. Therefore, the levels found for cadmium contamination may well have an additive effect, since cadmium and lead were found to coexist in most of the tested samples.

Arsenic can be considered as posing no significant threat to public health as a food contaminant in the study population as arsenic was not found in substantial quantities except in few outlier samples.

Forty two percent of study population were having detectable level of lead ranging from 1.7 (extrapolated) to 8.8 μg/dL with a mean of 3.0 μg/dL + SD 1.7.

The findings of this study would argue strongly towards derivation and implementation of policies that reduce the availability of food contaminated with lead. Adverse impacts of chronic low dose exposure to cadmium cannot be ruled out as this may enhance lead toxicity through a synergistic effect. These adverse outcomes are especially of concern in children and women of childbearing age.

The conclusions from this analysis will provide strong evidence of chronic low dose exposure to lead and cadmium through the diet. This may be associated with a number of possible adverse health outcomes, as it has been found elsewhere associated with lowering of GFR, hypertension, Low IQ, neoplasms and low birth weight. The described exposures may provide a vital framework for a series of science-based strategies and interventions that can be implemented to targeting better public health outcomes.

Following recommendations can be made on the evidence generated from this study. The study generated strong evidence of adverse health impacts from the dietary habits of the study population and since some of the possible contaminants of concern to human health are found in considerable concentrations in their food, two broad categories of strategies would need to be considered.

(1). Strategies to lower the exposure of lead and cadmium through food are strongly advocated. Possible strategies may include encouraging consumption of more food of animal origin (meat or fish), reducing availability of heavily contaminated food, identifying agronomic practices that can reduce heavy metal levels in food etc. It is pertinent to note that some earlier findings suggesting high levels of some heavy metals in inland fish have led residents in area to avoid consuming inland fish, but a number of other studies including our study shows that inland fish is not a significant source of the studied contaminants. Therefore, a programme targeting increased consumption of inland fish coupled with enhanced availability and regular monitoring of fish for possible contaminants is strongly suggested. In addition, availability of other popular meats like chicken may be promoted among this population. In summary, a sound evidence based health education programme is required to reduce confusion and increase clarity of these messages to people living in these areas.

(2) Strategies to prioritize follow up research. It is noted that a large number of studies have been undertaken utilizing considerable resources, but no priority has been given to estimate the exposure assessment of potential contaminants of concern, project their adverse health effects and derive programmes to reduce or eliminate the sources of such contamination. Such strategies might help to prevent the projected adverse health outcomes such as the full blown irreversible renal damage prevalent in several regions of Sri Lanka today.

It is proposed that future studies continue to focus on this trend in food contaminants in the area and corresponding lead and cadmium levels in suitable biological samples of the study population coupled with suitable biomarkers to detect early kidney injury. In addition, chronic lead toxicity can be evaluated in the population by testing the bone lead in vivo using x-ray fluoroscopy technique. A cohort study on the study population to monitor their exposure to these contaminants and their effects using suitable biomarkers and other methods is also suggested. We also suggest that regular testing of blood lead levels and bone lead levels coupled with sensitive biomarkers to identify early stages of kidney damage be included in future screening programmes in contrast to current screening strategies which can only detect patients with irreversible kidney damage. Lead exposure assessment is proposed to be included in patient management protocols especially for CKD cases and for patients with neurological impairments. Cadmium taken up by human body is mostly deposited in the kidneys and liver and therefore testing for cadmium in these tissues may further offer insights to levels of chronic exposure and health consequences thereof. Strategies for possible lowering of identified contaminants should also be explored.

## Abbreviations

AOAC: Association of Official Analytical Chemists
BMD: Benchmark dose
BMDL: percentile lower confidence limit of the benchmark dose
BW or bw: Body weight
CKD EPI: *Chronic Kidney Disease* Epidemiology Collaboration
CONTAM: European Food Safety Authority panel on Contaminants in the Food Chain
EFSA: European Food Safety Authority
FFQ: Food frequency questionnaire
GFR: Glomerular Filtration Rate
GN: Grama Niladhari
ICP MS: Inductively Coupled Plasma Mass Spectrometry
IQ: Intelligence Quotient
JEFCA: Joint WHO/FAO committee on food additives
LBW: Low birth weight
LOQ: Limit of quantification
MOH: Medical Officer of Health
PTWI: Provisional Tolerable Weekly Intake
SD: Standard Deviation
USEPA: United State Environmental Protection Agency
WHO GEMS: World Health Organization General Environment Management System
WHO: World Health Organization
